# Editorial: Zebrafish Models for Human Disease Studies

**DOI:** 10.3389/fcell.2022.861941

**Published:** 2022-03-11

**Authors:** Liqing Zang, Vincenzo Torraca, Yasuhito Shimada, Norihiro Nishimura

**Affiliations:** ^1^ Graduate School of Regional Innovation Studies, Mie University, Tsu, Japan; ^2^ London School of Hygiene and Tropical Medicine, University of London, London, United Kingdom; ^3^ School of Life Sciences, University of Westminster, London, United Kingdom; ^4^ Department of Integrative Pharmacology, Mie University Graduate School of Medicine, Tsu, Japan

**Keywords:** zebrafish, human disease, animal model, developmental biology, biomedical discovery

Zebrafish are an attractive vertebrate model organism for biomedical discovery (Tavares and Santos Lopes, 2013). The advantages of using zebrafish are well known and include cost-effectiveness, high fecundity, short generation time, external development, transparency of embryonic stages, and ease of genome manipulation. These features have provided investigators with a vertebrate model with unprecedented potential for the live imaging of biological processes (Okuda and Hogan, 2020) and genetic and drug screenings (Shah et al., 2015; Lam and Peterson, 2019).

Zebrafish models have been used in developmental biology and embryogenesis (Briggs, 2002; Mathavan et al., 2005). They have been increasingly used to investigate human diseases in the last 2 decades due to the high degree of genetic, anatomical, and physiological similarities to humans (Dooley and Zon, 2000; Adamson et al., 2018). Over 80% of disease-causing human proteins have an orthologue in zebrafish, and the publishing of the zebrafish reference genome in 2013 accelerated disease modelling in this organism. Consequently, our understanding of disease mechanisms and the development of new medical treatments have expanded. Notably, new therapeutic targets and molecules have been identified using zebrafish, which are now being considered for human trials or are awaiting clinical applications. Nevertheless, more zebrafish models are needed to broaden our understanding of human diseases.

The current research topic in *Frontiers of Cell and Developmental Biology* includes 35 original and review articles from 224 authors, containing a wide range of examples on how zebrafish contribute as an animal model to our understanding of various human diseases. The collection encompasses different areas of investigation: as further detailed by the sections below.

## In-Depth Exploitation of Established Zebrafish Models

Several authors have shed light on the contribution of established zebrafish disease models to recent and ongoing research efforts. A total of 13 review articles and 11 original articles in this research area have been accepted. Review articles provide an up-to-date overview of different zebrafish models for human diseases, including epilepsy (Chou et al.), osteoporosis (Rosa et al.), amyotrophic lateral sclerosis (ALS) (Asakawa et al.), inflammation (Xie et al.), atherosclerosis (Tang et al.), autism spectrum disorder (James et al.), heart failure (Narumanchi et al.), type 2 diabetes mellitus (Salehpour et al.), sensorineural hearing loss (Holmgren and Sheets), enteric nervous system disease (Kuil et al.), and cancer research (Miao et al.; Kobar et al.). At the time of this writing, the most viewed article titled *Modeling Inflammation in Zebrafish for the Development of Anti-Inflammatory Drugs* by Xie et al. summarised various inflammatory disease models proposed by zebrafish researchers and described inflammatory processes induced by wounding, exposure to chemicals, or mutations. These models were used to identify the molecular mechanisms underlying the inflammatory response and anti-inflammatory drugs. Asakawa et al. described the skeletal neuromuscular system of zebrafish larvae and introduced a recently developed optogenetic zebrafish model to study ALS. In this model, illumination can be used to control oligomerisation, phase transition, and aggregation of the ALS-associated DNA/RNA-binding protein TDP-43. In addition, zebrafish have emerged as a powerful model for heart disease studies. Abnormal cardiac structure and/or function (impaired blood flow, contractility, or relaxation) in zebrafish can be defined as heart failure. Narumanchi et al. reviewed heart failure zebrafish models established by genetic manipulation (morpholino and TALEN/CRISPR-Cas9-mediated knockdown or knockout) or drug treatment (such as aristolochic acid, isoproterenol, and streptozocin). Interestingly, zebrafish can repair and restore the cardiac function of a failing heart. Therefore, determining the molecular mechanisms underlying the regenerative ability of zebrafish will hopefully provide significant insights to advance the treatment of human heart failure in the years to come.

Among the original research articles, an interesting one titled *Live Imaging of Heart Injury in Larval Zebrafish Reveals a Multi-Stage Model of Neutrophil and Macrophage Migration* by Kaveh et al. has attracted considerable attention. The authors mapped the migration of immune cells (neutrophils and macrophages) from the caudal haematopoietic tissue via the blood or vasculature to the injury site using a larval zebrafish model of cardiac injury and the archetypal tail fin injury model. The finding could pave the way for future studies examining tissue injury and inflammation. In the drug discovery and safety fields, Westhoff et al. performed a large-scale screening of the kidney-specific toxicity of approved drugs using a *Tg(wt1b:EGFP)* zebrafish line, which could provide a unique platform for *in vivo* large-scale assessment of organ-specific developmental toxicity or other biomedical applications.

## Novel Human Disease Models

Two articles reported the use of novel zebrafish models for human pseudoxanthoma elasticum (PXE), and mitochondrial membrane protein-associated neurodegeneration (MPAN). PXE is a rare genetic disease resulting from the dysfunction of ATP-binding cassette subfamily C member 6 (ABCC6). Czimer et al. found that zebrafish deficient in *abcc6a* showed vertebral calcification defects and ectopic calcification foci in the soft tissues, thereby replicating the symptoms of human PXE. In a study titled *The Downregulation of c19orf12 Negatively Affects Neuronal and Musculature Development in Zebrafish Embryos*, Mignani et al. described a new zebrafish model for MPAN generated via *c19orf12* knockdown using a specific ATG-blocking morpholino. The embryos exhibited morphological defects, such as unsettled brain morphology with small heads and eyes, which were consistent with the clinical features of MPAN.

## New Technologies for Developing Human Disease Models

Several studies have experimented with novel technologies for the better utilisation of zebrafish models for human diseases. Silva et al. developed an easy-to-handle dietary cholesterol-based *in vivo* assay that allows the screening of immunomodulatory therapeutics using the transgenic strain *Tg(Lyz:NTR-mCherry)* ^*sh260*^ (Buchan et al., 2019). High cholesterol diet-induced acute intestinal inflammation in larval zebrafish can be monitored by this assay, which can facilitate and accelerate drug discovery efforts for human inflammatory bowel diseases.

## Developmental Biology

Zebrafish have traditionally been used as a developmental biology model. In this research topic, Piedade et al. reported the role of cadherin-related family member 1a (*Cdhr1a*; encoding a homologue of mammalian CDHR1) in early photoreceptor development in zebrafish embryos and further demonstrated that *Cdhr1a* is directly regulated by the ubiquitin-proteasome system via interaction with *Siah1*. Peña et al. investigated the deficiency of two ohnologues of the GATA-binding protein 2 (GATA2) genes (*gata2a* and *gata2b*) to define their roles in developmental haematopoiesis. It was concluded that each ohnologue may have specific roles at different stages of developmental myelopoiesis and in the emergence of haematopoietic stem and progenitor cells.

## CONCLUDING REMARKS

In summary, this research topic gathered a wide spectrum of high-quality articles demonstrating the impact that zebrafish research has on our understanding of human diseases. We acknowledge all authors who contributed to this research topic, and we hope that this collection will serve to inspire the community by providing insights into the potential of using zebrafish models to study human diseases.
